# News Items

**Published:** 1917-03

**Authors:** 


### News Items.

The next annual convention of the Southern Sociological Congress will be held at Blue Ridge, N. C., July 30th to August 3d.

---

Dr. C. W. Winckel, Public Health Officer of the Island of Java, Dutch East Indies, has been spending a few days in Dallas, seeking information to be applied in bettering health conditions in Java. Most of his investigations are being made in the South. From Dallas he will go to New Orleans, then to Cuba and Panama.

Dr. Winckel says that little health work has been done among the 30,000,000 natives inhabiting Java, but the Dutch government is planning a vigorous campaign to better health conditions.

---

Irving has been selected as a health demonstration city. It was voted unanimously at a meeting in February to begin the health work at once.

---

Ennis is to have a $20,000 sanitarium. The institution is being backed by local capital, and will be modern in every respect.

---

Bids have been opened for the construction of a new sanitarium at Lubbock, to be erected at a cost of approximately $75,000. It will be known as the Ponton Sanitarium.

---

Under the management of Drs. D. M. Cummins, W. A. Kimbrough and L. F. Taylor, assisted by two trained nurses, a first-class sanitarium has been opened in Haskell.

---

The handsome new Karbach Hospital, situated at Schertz, has just been opened. This building is of reinforced concrete, two-story with a roof garden, and modern in every respect. The hospital is owned and operated by Miss Cora Karbach.

---

Volume I, No. 1, of the new *Journal of Urology* is just off the press. Dr. Hugh H. Young of Johns Hopkins is the editor. The *Journal* will be issued bimonthly beginning with February issue, 1917. Subscription is by volume only, and not by the year; price in United States, $5.00. Each volume will consist of approximately 600 pages. The *Journal* is published by Wilkins & Wilkins, of Baltimore, and all subscriptions should be sent to them, care of Charles C. Thomas, Circulation Manager. The *Journal* has been founded with the idea that it shall become the archives in the United States for papers dealing with the urinary tract and correlated subjects. The personnel of the editorial board is an assurance of the high standards which the *Journal* will maintain.

Dr. W. M. Stell, for thirty years a practicing physician in Mexico, who has repeatedly been mentioned in the dispatches as having been killed, or whose fate was unknown, writes his father, Dr. W. W. Stell, of Paris, that he is now located in El Paso.

Dr. Doak, of Taylor, is in New York completing a special course in general surgery at the New York Postgraduate School and Hospital. He is also taking other special courses in diseases of women and general medicine and diseases of children. Dr. Doak will likely remain in New York several weeks.

Dr. T. P. Davis, of Terrell, while driving his automobile across the Texas & Pacific track, on February 15th, was struck by e passenger train and severely injured. The car was badly damaged and Dr. Davis was thrown out and rendered unconscious.

Dr. F. S. White, of San Antonio, has just returned from a three weeks' stay in New Orleans, where he took Mrs. White for an operation.

Dr. S. W. Lytal, of Quinlan, has just finished a four weeks' study in review and clinical work at Texas Christian University, Fort Worth.

Dr. J. S. Wilkins, of Paducah, is in Rochester, Minnesota, with his brother, Dr. T. O. Watkins, who is quite ill. While there Dr. J. S. Watkins is taking advantage of the clinics.

Dr. T. J. Johnson and Miss Nannie Asbury, of Rosebud, were married at Marlin February 22d.

---

### Army Medical Corps Examinations.

The Surgeon General of the Army announces that preliminary examinations for appointment of first lieutenants in the army medical corps will be held at convenient points the first Monday in each month. Full information concerning these examinations can be procured upon application to the "Surgeon General, U. S. Army, Washington, D. C."

The essential requirements to secure an invitation are that the applicant shall be a citizen of the United States, shall be between 22 and 32 years of age at the time of commission at the close of the army medical school, a graduate of a medical school legally authorized to confer the degree of doctor of medicine, shall be of good moral character and habits, and shall have had at least one year's hospital training as interne after graduation.

Graduate physicians who are serving their interneship and who meet the other requirements can be examined for appointment with the understanding that they will complete the required post-graduate hospital interneship before coming to the army medical school.

Those who qualify at their preliminary examination and complete their hospital interneship by July 1st will be ordered to the army medical school for the special session of the school commencing July 9th. The regular session of the school will open on October 1st.

In order to perfect all arrangements for the examination, applications should be completed at the earliest practicable date.

There are at present 230 vacancies in the army medical corps. After July 1st, there will be 222 additional vacancies.

### Deaths.

Dr. Samuel R. Graves died at the home of his son, S. R. Graves, of Lockhart, February 24th.

Dr. J. W. McCulloch, of Argyle, died February 17th after a lingering illness of tuberculosis.

Dr. G. T. Parks died at his residence in Lancaster on February 24th, after an illness of several months. Dr. Parks was born and reared near Lancaster, and had been engaged in the practice of his profession at Lancaster for thirty years.

Dr. D. F. Ashby, of Paris, was shot and fatally wounded at his home on February 23d. He died about forty minutes later.

Dr. W. N. Bunkley died at his home in Stamford February 20th.

Dr. R. R. White, Jr., Chief Surgeon of the Gulf, Colorado & Santa Fe Railroad, died at his home in Temple on March 2d of heart failure. He was sick only a day or two, and his death was a shock to his many friends and relatives.

